# Sarcopenia increases the risk of major organ or vessel invasion in patients with papillary thyroid cancer

**DOI:** 10.1038/s41598-022-08224-x

**Published:** 2022-03-10

**Authors:** Ja Kyung Yoon, Jung Hyun Yoon, Vivian Youngjean Park, Minah Lee, Jin Young Kwak

**Affiliations:** Department of Radiology and Research Institute of Radiological Science, Severance Hospital, Yonsei University College of Medicine, 50-1 Yonsei-ro, Seodaemun-gu, Seoul, 03722 Korea

**Keywords:** Prognostic markers, Thyroid cancer, Thyroid cancer

## Abstract

While sarcopenia is associated with poor overall survival and cancer-specific survival in solid cancer patients, the impact of sarcopenia on clinicopathologic features that can influence conventional papillary thyroid cancer (PTC) prognosis remains unclear. To investigate the impact of sarcopenia on aggressive clinicopathologic features in PTC patients, prospectively collected data on 305 patients who underwent surgery for PTC with preoperative staging ultrasonography and bioelectrical impedance analysis were retrospectively analyzed. Nine sarcopenia patients with preoperative sarcopenia showed more patients aged 55 or older (*p* = 0.022), higher male proportion (*p* < 0.001), lower body-mass index (*p* = 0.015), higher incidence of major organ or vessel invasion (*p* = 0.001), higher T stage (*p* = 0.002), higher TNM stage (*p* = 0.007), and more tumor recurrence (*p* = 0.023) compared to the non-sarcopenia patients. Unadjusted and adjusted logistic regression analyses showed that sarcopenia (odds ratio (OR) 9.936, 95% confidence interval (CI) 2.052–48.111, *p* = 0.004), tumor size (OR 1.048, 95% CI 1.005–1.093, *p* = 0.027), and tumor multiplicity (OR 3.323, 95% CI 1.048–10.534, *p* = 0.041) significantly increased the risk of T4 cancer. Sarcopenia patients showed significantly lower disease-free survival probability compared to non-sarcopenia patients. Therefore, preoperative sarcopenia in PTC patients should raise clinical suspicion for a more locally advanced disease and direct appropriate management and careful follow-up.

## Introduction

Sarcopenia is defined as the age-related loss of skeletal muscle mass and function, and is associated with metabolic, physiologic, and functional impairments^1,2^. Clinically, sarcopenia is identified by low muscle strength as well as low muscle quantity and quality^2^. Such changes in body composition can impact an extensive variety of disease processes, such as cardiac disease, respiratory disease, as well as some malignancies^3–6^.

In oncology, severe sarcopenia may occur in cancer patients with profound weight loss, particularly of the skeletal muscle^7,8^. A high prevalence of sarcopenia has been reported in gastric cancer (57%), advanced hepatocellular carcinoma (HCC) (27.5%), and metastatic renal cell carcinoma (RCC) (29%)^9–11^. Sarcopenia has also been associated with poor overall survival and cancer-specific survival in breast cancer, HCC, RCC, and gastrointestinal tract cancer. In addition, sarcopenia has also been associated with increased chemotherapy toxicity and post-operative complications in various solid cancers^4,12–14^. However, sarcopenia has not been associated with poor survival in soft tissue sarcoma^14^, suggesting that these associations may vary according to the specific cancer type.

In thyroid cancers, only data on post-operative or post-treatment sarcopenia on patient prognosis have been reported. The DECISION trial showed a significant association between sorafenib and reduced muscle mass in advanced differentiated thyroid cancer^15^. In addition, a recent study showed that low thyroid stimulating hormone (TSH) levels after total thyroidectomy in PTC patients was associated with low grip strength, a parameter for assessing sarcopenia^16^. However, past studies have not explained whether preoperative sarcopenia can act as an aggressive clinicopathologic feature that may potentially affect the prognosis of conventional papillary thyroid cancer (PTC).

Therefore, this study aimed to investigate the potential implications of preoperative sarcopenia on aggressive clinicopathologic features in PTC patients.

## Results

### Baseline characteristics

Baseline clinicopathologic characteristics of the 305 patients with conventional PTC are summarized in Table 1. The median age was 43.0 years (interquartile range (IQR), 33.0–53.0 years) with 231 women (median age 43.0 years [IQR, 33.0–53.0 years]) and 74 men (median age, 41.0 years [IQR, 32.0–54.3 years])). Based on the bioelectrical impedance analysis (BIA) measurements of skeletal muscle index (SMI), nine patients (3.0%) were diagnosed with sarcopenia preoperatively. There were 234 patients (76.6%) with no or minimal extrathyroidal extension (ETE), 55 patients (18.0%) with gross strap muscle invasion, and 16 patients (5.3%) with major organ invasion involving the trachea, esophagus, recurrent laryngeal nerve, or major vessels. There were no cases of distant metastasis. The overall mean and standard deviation of body mass index (BMI) was 23.5 ± 3.4 kg/m^2^ [(range, 16.4–33.9 kg/m^2^) and there were 92 obese patients (30.2%). The median primary tumor size was 14.0 mm (IQR, 12.0–19.0 mm), ranging from 11.0 mm to 130.0 mm. The median disease-free survival was 63.4 months (IQR, 59.5–71.3 months), ranging from 3.1 months to 84.6 months. Tumor recurrence occurred in a total of five patients (1.65%).

**Table 1:** Clinicopathologic characteristics of the study population.

Characteristic	All patients
Age (years)*	43.0 (33.0, 53.0)
≥ 55 years old	64 (21.0%)
Female	231 (75.7%)
Weight (kg)^†^	62.5 ± 11.6
Height (cm)^†^	162.9 ± 8.1
BMI (kg/m^2^)^†^	23.5 ± 3.4
Obesity (BMI ≥ 25)	92 (30.2%)
Sarcopenia	9 (3.0%)
Tumor size (mm)*	14.0 (12.0, 19.0)
**Extrathyroidal extension (ETE)**	
No or minimal ETE	234 (76.7%)
Gross strap muscle invasion	55 (18.0%)
Major organ or vessel invasion	16 (5.2%)
Tumor multiplicity	121 (39.7%)
LN metastasis	182 (59.7%)
Distant metastasis	0 (0.0%)
**T stage (AJCC 8th edition) **^35^	
T1	200 (65.6%)
T2	31 (10.2%)
T3	58 (19.0%)
T4a	16 (5.2%)
**TNM staging (AJCC 8th edition)**^35^	
Stage I	266 (87.2%)
Stage II	35 (11.5%)
Stage III	4 (1.3%)
Stage IV	0 (0.0%)
Tumor recurrence	5 (1.6%)
Disease-free survival (months)*	63.4 (59.5, 71.3)

Data are presented as number of patients (percent) unless otherwise specified. *Data are presented as median (1st quartile, 3rd quartile). ^†^Data are presented as mean ± standard deviation. *BMI,* body mass index; *AJCC*, American Joint Committee on Cancer; *LN*, lymph node.

### Clinicopathologic features of PTC in the sarcopenia group

**Figure 1:**
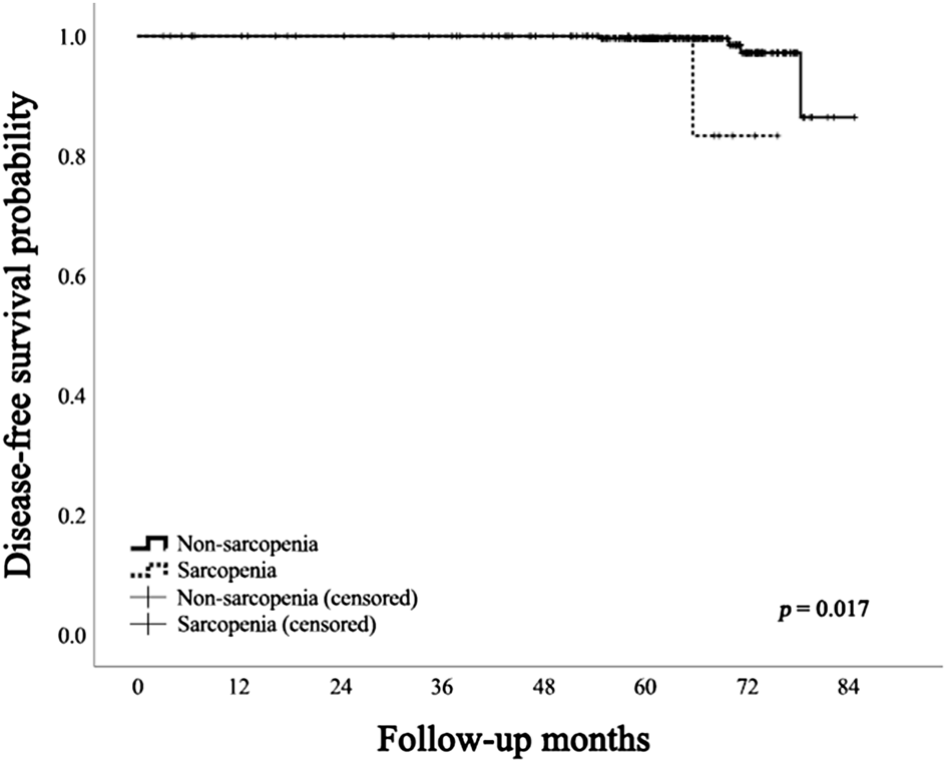
Disease-free survival probabilities of the sarcopenia and non-sarcopenia groups. The disease-free survival probability was lower in the sarcopenia group compared to the non-sarcopenia group, with statistical significance.

Compared to the non-sarcopenia group, the sarcopenia group showed more patients older than 55 years of age (*p* = 0.022), higher male proportion (*p* < 0.001), lower BMI (*p* = 0.015), and more tumor recurrence (*p* = 0.023) (Table 2). The sarcopenia group showed more frequent major organ or vessel invasion (*p* = 0.001), high T stage (*p* = 0.002), and high overall TNM stage (*p* = 0.007) than the non-sarcopenia group. There were no significant differences in LN metastasis between the two groups. There was no significant difference in median disease-free survival between the two groups (non-sarcopenia 63.3 months versus sarcopenia 68.1 months, *p* = 0.425). The disease-free survival probability at 6th year was significantly lower in the sarcopenia group compared to the non-sarcopenia group (0.833 versus 0.972, *p* = 0.017) (Fig. 1). The four tumor recurrences from the non-sarcopenia group occurred at a median of 70.5 months, and the recurrence in the sarcopenia group occurred at 65.5 months.

**Table 2:** Clinicopathologic features of patients according to the presence of sarcopenia.

Characteristic	Non-sarcopenia (n = 296)	Sarcopenia (n = 9)	*p-*value
Age (years)*	42.5 (33.0, 52.0)	58.0 (30.0, 62.0)	0.392
≥ 55 years old	59 (19.9%)	5 (55.6%)	**0.022**
Male	66 (22.3%)	8 (88.9%)	** < 0.001**
BMI (kg/m^2^)^†^	23.6 ± 3.4	20.8 ± 2.2	**0.015**
Obesity (BMI ≥ 25)	92 (31.1%)	0 (0.0%)	0.062
Tumor size (mm)*	14.0 (12.0, 19.0)	18.0 (11.0, 25.5)	0.323
Range	11.0–130.0	11.0–43.0	
Tumor multiplicity	117 (39.5%)	5 (55.6%)	0.744
**Extrathyroidal extension (ETE)**			**0.001**
No or minimal	229 (77.4%)	5 (55.6%)	
Gross strap muscle invasion	54 (18.2%)	1 (11.1%)	
Major organ or vessel invasion	13 (4.4%)	3 (33.3%)	
**T stage (AJCC 8th edition)**^35^			**0.019**
T1	196 (66.2%)	4 (44.4%)	
T2	30 (10.1%)	1 (11.1%)	0.666
T3	57 (19.3%)	1 (11.1%)	0.893
T4a	13 (4.4%)	3 (33.3%)	**0.003**
LN metastasis	175 (59.1%)	0 (0.0%)	0.261
**TNM staging (AJCC 8th edition)**^35^			**0.003**
Stage I	261 (88.2%)	5 (55.6%)	
Stage II	32 (10.8%)	3 (33.3%)	
Stage III	3 (1.0%)	1 (11.1%)	
Tumor recurrence	4 (1.4%)	1 (11.1%)	**0.023**
Disease-free survival (months)*	63.3 (59.5, 71.3)	68.1 (60.4, 71.5)	0.425

Significant values are in bold.

Data are presented as number of patients (percent) unless otherwise specified. *Data are presented as median (1st quartile, 3rd quartile). ^†^Data are presented as mean ± standard deviation. *BMI,* body mass index; *AJCC*, American Joint Committee on Cancer; *LN*, lymph node.

### Unadjusted and adjusted logistic regression models between patient body composition and aggressive tumor features

Unadjusted logistic regression analysis between sarcopenia and various clinicopathologic features revealed that sarcopenia was significantly associated with major organ or vessel invasion (T4) (odds ratio (OR) 10.885, 95% confidence interval (CI) 2.445–48.453, *p* = 0.002) and high TNM stage (OR 12.208, 95% CI 1.142–130.552, *p* = 0.038) (Table 3). Sarcopenia was not significantly associated with high T stage (T3 or T4), lymph node (LN) metastasis, or tumor recurrence (Table 3).

**Table 3:** Unadjusted logistic regression analyses between sarcopenia and clinicopathologic features.

	Tumor multiplicity	Any ETE	Major organ or vessel invasion (T4)	LN metastasis	High T stage (T3 or T4)	High TNM stage (III or IV)	Tumor recurrence
**Sarcopenia**							
OR (95% CI)	1.224 (0.322–4.652)	2.734 (0.714–10.470)	10.885 (2.445–48.453)	2.42 (0.494–11.849)	2.583 (0.675–9.882)	12.208 (1.142–130.552)	9.125 (0.914–91.117)
*p*-value	0.767	0.142	**0.002**	0.273	0.166	**0.038**	0.060

Significant values are in bold.

*OR* odds ratio, *CI* confidence interval, *ETE* extrathyroidal extension, *LN* lymph node.

On the unadjusted logistic regression analysis for clinicopathologic features and major organ invasion, sarcopenia, tumor size, tumor multiplicity, and LN metastasis showed significant associations with major organ or vessel invasion (T4) (Table 4). On adjusted logistic regression including these variables, patients with sarcopenia (OR 9.936, 95% CI 2.052–48.111, *p* = 0.004), larger tumor size (OR 1.048, 95% CI 1.005–1.093, *p* = 0.027), and tumor multiplicity (OR 3.323, 95% CI 1.048–10.534, *p* = 0.041) showed significantly higher risk of major organ or vessel invasion (T4).

**Table 4:** Unadjusted and adjusted logistic regression analyses between clinicopathologic features and major organ or vessel invasion (T4).

	Unadjusted			Adjusted		
	OR	95% CI	*p*-value	OR	95% CI	*p*-value
Age	1.023	0.983–1.065	0.265	–	–	–
Male gender	1.950	0.684–5.561	0.212	–	–	–
Obesity	1.928	0.536–6.935	0.315	–	–	–
Sarcopenia	10.885	2.445–48.453	**0.002**	9.936	2.052–48.111	**0.004**
Tumor size	1.055	1.014–1.099	**0.009**	1.048	1.005–1.093	**0.027**
Tumor multiplicity	3.477	1.177–10.272	**0.024**	3.323	1.048–10.534	**0.041**
LN metastasis	5.042	1.125–22.593	**0.035**	3.545	0.757–16.599	0.108

Significant values are in bold.

*OR* odds ratio, *CI* confidence interval, *LN* lymph node.

Unadjusted logistic regression analysis for clinicopathologic features and high TNM stage showed that age (OR 1.157, 95% CI 1.032–1.299, *p* = 0.013) and sarcopenia (OR 12.208, 95% CI 1.142–130.552, *p* = 0.038) were significantly associated with high TNM stage. However, adjusted regression analysis revealed that only older age (OR 1.148, 95% CI 1.018–1.294, *p* = 0.024) and not sarcopenia (OR 6.099, 95% CI 0.478–77.874, *p* = 0.164) was a significant risk factor for high TNM stage (Supplementary Information 1).

## Discussion

In this study, sarcopenia was a significant risk factor for major organ or vessel invasion (or T4 cancer) in PTC, and consequently a significant risk factor for more locally advanced disease. Preoperative sarcopenia significantly decreased the disease-free survival probability in PTC.

Although significant associations between sarcopenia and adverse cancer outcomes have been suggested^4^, published literature on the link between sarcopenia and PTC have not been clearly elucidated. Available previous studies suggest a major role of pro-inflammatory and anti-inflammatory cytokines in sarcopenia which may also influence the aggressiveness of PTC^17^. Serum interleukin 6 (IL-6), which is increased in cancer-caused sarcopenia, is found in high levels in PTC patients and showed a significant positive correlation with larger tumor size and ETE^18^. In addition, IL-6 mRNA was a significant prognostic factor for overall survival but not disease-free survival in PTC patients^18^. In addition, another study suggested that some PTC cells may be able to escape the suppression of cellular proliferation and invasiveness of IL-1β, a cytokine which is increased in sarcopenia^19^. This suggests that escaping the antitumor effect of IL-1β may be a step towards anaplastic change that could result in a more aggressive thyroid cancer^19,20^. These findings suggest an immunologic basis for sarcopenia and PTC aggressiveness^20^.

Preoperative sarcopenia significantly lowered disease-free survival probability, but it did not significantly increase the risk of tumor recurrence. This apparent discrepancy may indicate that sarcopenia is a risk factor for earlier tumor recurrence rather than the risk of tumor recurrence itself. Indeed, the median disease-free survival of tumor recurrence in the sarcopenia patient occurred earlier than the that of the non-sarcopenia patients, but without statistical significance due to the low number of tumor recurrences. This inference may be further supported by the fact that the prognostic impact of gross strap muscle invasion in PTC has been challenged in recent years^21–24^. Despite the AJCC 8th TNM staging criteria, these findings may be attributed to advancements in surgical techniques as well as complete en bloc resection, even in cases with advanced ETE^25–27^. These findings may indicate that at-risk PTC patients for tumor recurrence diagnosed with preoperative sarcopenia may require a more continuous post-operative follow-up to detect earlier tumor recurrence.

Preoperative sarcopenia was not significantly associated with LN metastasis, which may be the reason for the lack of significant association between high TNM stage on adjusted logistic regression analysis, despite a significant association on unadjusted analysis. In addition, the association between sarcopenia and LN metastasis seems to vary according to the type of cancer^28–31^. Sarcopenia has been shown to increase the risk of LN metastasis in colorectal cancer and advanced urothelial carcinoma, but not in resectable bile duct cancer or recurrent pancreas adenocarcinoma^28–31^. Our study suggests that sarcopenia is not significantly associated with the risk of LN metastasis in PTC.

In our study, body composition analysis and identification of preoperative sarcopenia was done using the BIA, an affordable, widely available, and portable instrument with reproducible results^2,32^. Unlike other malignancies in which abdomen and pelvic CT scans are often included during routine staging work-up, measuring SMI on CT scans to assess sarcopenia in thyroid cancer may unnecessarily burden patients with more radiation exposure. Alternatively, BIA may be an easier, more affordable tool to diagnose sarcopenia in PTC, as demonstrated in our study. In addition, the assessment of sarcopenia may be of importance in treatment decisions for advanced or refractory thyroid cancer because sarcopenia is considered a contraindication or discouraging factor in tyrosine kinase inhibitor treatment^15,33^.

There are several limitations to this study. First, an inherent bias owing to the retrospective analysis of prospectively obtained data was inevitable. Second, the very low incidence of not only preoperative sarcopenia, but also of tumor recurrence in our study is a critical limitation. In addition, the impact of sarcopenia on the overall survival of PTC patients could not be evaluated due to no deaths during follow-up of our study population. Lastly, body composition analysis by BIA may yield inconsistent or discrepant results depending on different instrument brands, as well as different population characteristics^2^. Therefore, we utilized cutoff values previously obtained with an identical equipment in young, healthy Korean subjects^34^. However, the findings of this study may be limited to Korean patients. Further research that includes patients from more diverse populations may help confirm the impact of sarcopenia on the outcomes and clinicopathologic features of PTC patients.

In conclusion, preoperative sarcopenia is significantly associated with a higher risk of major organ invasion in conventional PTC, which significantly decreased disease-free survival probability. Preoperative diagnosis of sarcopenia in PTC patients should raise clinical, radiological, and surgical suspicion for a more locally advanced disease and direct appropriate management and follow-up.

## Methods

This prospective study was approved by the Severance Hospital Institutional Review Board, and was conducted in accordance with the Declaration of Helsinki as revised in 2013. Informed consent was obtained from all patients enrolled in this study.

### Study population

**Figure 2:**
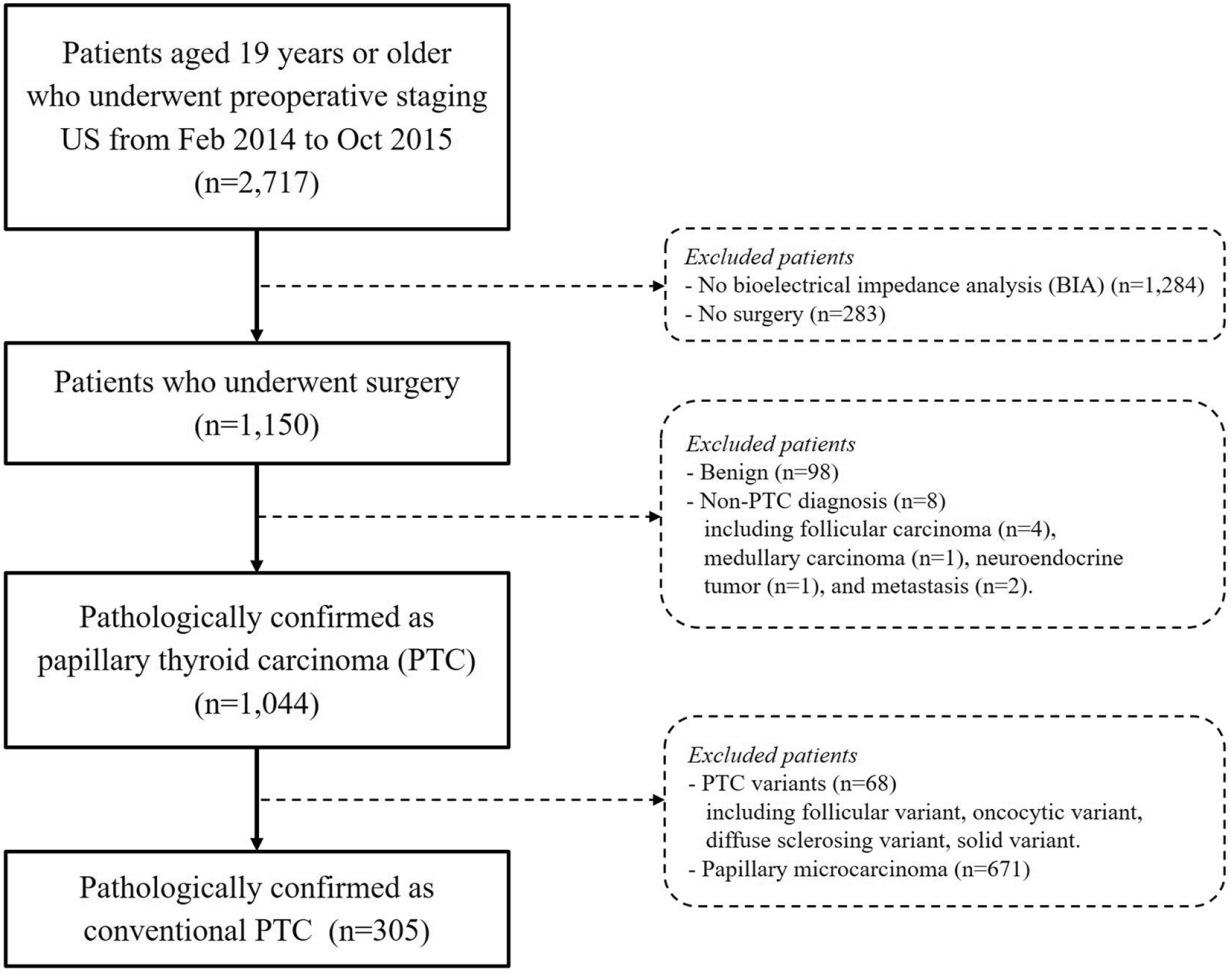
Patient selection diagram. A total of 2,717 patients were enrolled. Patients who did not undergo preoperative bioelectrical impedance analysis (BIA) or thyroid surgery were excluded. Only pathologic diagnosis of conventional PTC were included, resulting in a total of 305 patients eligible for final analysis.

From February 2014 to October 2015, a total of 2,717 patients aged 19 years or older underwent preoperative staging ultrasonography (US) at our institution. Patients who did not undergo preoperative BIA (n = 1,284) or thyroid surgery (n = 283) were excluded. Patients with benign pathology (including diffuse hyperplasia, adenomatous hyperplasia, follicular adenoma, Hurthle cell adenoma, Hashimoto’s thyroiditis, lymphocytic thyroiditis, Grave’s disease, hyalinizing trabecular tumor and branchial cleft cyst anomaly type II) (n = 98), PTC variants (follicular variant, oncocytic variant, diffuse sclerosing variant, and solid variant PTC) (n = 68), follicular carcinoma (n = 4), medullary carcinoma (n = 1), neuroendocrine tumor (n = 1), metastatic adenocarcinoma (n = 2), as well as papillary microcarcinoma (n = 671) were excluded. A total of 305 patients with a pathologically confirmed diagnosis of conventional PTC were eligible for final analysis (Fig. 2). Disease-free survival was defined as the time interval from the date of diagnosis to the last follow-up visit or the date of tumor recurrence.

### Preoperative staging ultrasonography (US)

Preoperative staging US was performed by one of 17 radiologists (12 fellows with 1 or 2 years of experience and 5 faculties with 6 to 18 years of experience in thyroid US) using a 5–12 MHz linear transducer (iU22, Philips Medical Systems, Bothell, WA). Primary tumor and cervical LNs were evaluated and reviewed based on the 8th AJCC TNM classification^35^. Any LN with at least one suspicious US feature (focal or diffuse hyperechogenicity, presence of internal calcification, cystic change, round shape, and chaotic or peripheral vascularity on Doppler US) was considered pathologic and was subsequently confirmed by either US-FNA or surgery.

### Bioelectrical impedance analysis (BIA)

All included patients underwent preoperative BIA. Multifrequency bioimpedance data was measured using In-Body 720 (Biospace, Seoul, South Korea) in a standardized manner for all patients who underwent thyroid surgery. In-Body 720 employs a direct segmental multi-frequency bioelectrical impedance (MF-BIA) analysis method using a tetrapolar 8-point tactile electrode system with 30 impedance measurements taken at 6 frequencies (1 kHz, 5 kHz, 50 kHz, 250 kHz, 500 kHz, 1000 kHz) and reactance evaluated by 15 impedance measurements at 3 frequencies (5 kHz, 50 kHz, 250 kHz) for each of the 5 body segments (right arm, left arm, trunk, right leg, and left leg). All measurements were carried out prior to surgery in all patients. Body fat mass, body fat percentage, muscle mass, skeletal muscle mass, visceral fat surface, waist-hip ratio, and basal metabolic rate were measured to assess body composition. Furthermore, the outer circumference and fat thickness of various body compartments (neck, chest, abdomen, hip, arm, and thigh) were measured. Finally, the SMI (skeletal muscle mass/height^2^) and BMI of each patient was analyzed using the aforementioned parameters. Sarcopenia was diagnosed according to sex-specific cutoff values (two standard deviations below the mean) for SMI that were suggested in a previous study of a healthy Korean population^34^. Obesity was categorized according to the BMI as follows: underweight (< 18.5 kg/m^2^), normal weight (18.5–22.9 kg/m^2^), overweight (23.0–24.9 kg/m^2^), obesity (25.0–29.9 kg/m^2^) and severe obesity (≥ 30 kg/m^2^) according to the World Health Organization guidelines for Asians^36^.

### Thyroid surgery and pathologic diagnosis

All total thyroidectomy at our institution routinely included bilateral central compartment LN dissection (CCND), which included the paratracheal, pretracheal, and prelaryngeal LNs. All hemithyroidectomy surgeries routinely included unilateral CCND. Selective lateral compartment LN dissection was performed only when lateral LN metastasis was suspected on either preoperative US and US-FNA, or during intraoperative observations. The lateral compartment included level II, III, IV, and V LNs. Pathologic tumor size, tumor multiplicity, ETE, and the presence of central and/or lateral LN metastasis on final surgical pathology reports were reviewed for analysis. TNM staging was performed according to the AJCC 8th staging criteria^35^. T staging was done as follows: (1) T1, tumor size smaller than 20 mm in greatest dimension limited to the thyroid; (2) T2, tumor size larger than 20 mm but equal to or smaller than 40 mm in greatest dimension limited to the thyroid; (3) T3a, tumor size larger than 40 mm but limited to the thyroid; (4) T3b, tumor of any size with gross strap muscle invasion; (5) T4, tumor of any size with invasion of major neck structures such as the larynx, trachea, esophagus, recurrent laryngeal nerve, prevertebral fascia or encasing major vessels. N staging was done as follow: (1) N0, no evidence of regional LN metastasis; (2) N1a, metastasis to level VI or VII (pretracheal, paratracheal, or prelaryngeal/Delphian, or upper mediastinal); (3) N1b, metastasis to unilateral, bilateral, or contralateral lateral neck LNs (levels I, II, III, IV, or V) or retropharyngeal LNs. T3 and T4 were considered high T stages, and the overall TNM stages III and IV were considered as high TNM stages.

### Statistical analysis

All statistical analyses were performed with commercial software (IBM SPSS Statistics, version 25.0; IBM Corp., Armonk, NY). The independent t-test or Mann–Whitney U test was performed to compare clinical and pathological variables for parametric and non-parametric continuous variables, respectively. Categorical variables were compared using the chi square test or Fisher’s exact test. Logistic regression analyses were performed to estimate unadjusted ORs with 95% CIs to elucidate the association between sarcopenia and aggressive tumor features (such as tumor multiplicity, ETE, LN metastasis, T stage, TNM stage, and tumor recurrence). Unadjusted logistic regression analysis was performed to explore the potential implications of sarcopenia on those aggressive tumor features. For variables that were significantly associated with sarcopenia on unadjusted analysis, a subsequent adjusted logistic regression analysis was performed that included the other clinicopathologic variables to obtain ORs and 95% CIs. The Kaplan–Meier analysis with log-rank test was performed to compare the disease-free survival between the sarcopenia group and non-sarcopenia group. A *p-*value of less than 0.05 was considered statistically significant.

## Supplementary Information

Supplementary Information.
