# Supplementary material for: Sarcopenia increases the risk of major organ or vessel invasion in patients with papillary thyroid cancer
Source: Sci Rep. 2022 Mar 10;12:4233. doi: 10.1038/s41598-022-08224-x (PMC8913816; doi:10.1038/s41598-022-08224-x)
Supplement: Supplementary file 1 — Supplementary Information. [file 41598_2022_8224_MOESM1_ESM.pdf]

Sarcopenia increases the risk of major organ or vessel invasion in patients with papillary thyroid cancer

Ja Kyung Yoon, M.D., Ph.D[1], Jung Hyun Yoon, M.D., Ph.D[1], Vivian Youngjean Park, M.D., Ph.D[1], Minah Lee, M.D.[1], Jin Young Kwak, M.D., Ph.D[1*]

[1]Department of Radiology and Research Institute of Radiological Science, Severance Hospital, Yonsei University College of Medicine

Supplementary Information 1. Unadjusted and adjusted logistic regression analyses between clinicopathologic features and high TNM stage (stage III and IV).

	**Unadjusted**			**Adjusted**		
	OR	95% CI	*p*-value	OR	95% CI	*p*-value
Age	1.157	1.032 - 1.299	**0.013**	1.148	1.018 - 1.294	**0.024**
Male gender	1.041	0.107 - 10.163	0.972	-	-	-
Obesity	0.769	0.079 - 7.494	0.821	-	-	-
Sarcopenia	12.208	1.142 - 130.552	**0.038**	6.099	0.478 - 77.874	0.164
Tumor size	0.996	0.890 - 1.114	0.941	-	-	-
Tumor multiplicity	0.503	0.052 - 4.891	0.554	-	-	-
LN metastasis	2.045	0.210 - 19.887	0.538	-	-	-

OR, odds ratio; *CI,* confidence interval; *LN,* lymph node.
